# Renal-inferior vena cava fistula complicating laparoscopic cholecystectomy causing heart failure repaired endovascularly, a case report and literature review

**DOI:** 10.1016/j.ijscr.2023.107873

**Published:** 2023-01-04

**Authors:** Ahmed Almumtin, Mohammed Dahman, Bassam Khalil, Mitham Alabduljabbar, Elaf Almusahel, Samer Koussayer

**Affiliations:** a KFSH-RC, Saudi Arabia; b King Saud University, Riyadh, Saudi Arabia; c King Abdulaziz Medical City, Saudi Arabia

**Keywords:** IVC, inferior vena cava, CTA, computed tomographic angiography, AVF, arteriovenous fistula, EF, ejection fraction, LV, left ventricle, HOHF, high output heart failure, BP, blood pressure, RAVF, renal arterial venous fistula, Renal-caval fistula, Iatrogenic fistula, Laparoscopic cholecystectomy, Arteriovenous fistula, Endovascular repair, Case report

## Abstract

**Introduction:**

Renal-caval Arterio-venous fistulas are rare entity which can be acquired, idiopathic or congenital. Laparoscopic cholecystectomy complicated by arteriovenous fistula formation is extremely rare and often go unnoticed. High output heart failure can occur as a consequence of such high flow fistulas. Repair can be done through open or endovascular approach with the latter being effective and less invasive. Repair can result in resolution of symptoms and improvement of heart function.

**Case presentation:**

We report a 43-year old female who developed an iatrogenic renal-caval fistula following laparoscopic cholecystectomy, that was complicated by intraoperative bleeding. She presented with worsening high output cardiac failure a year post-operative. Due to past history of Cor-triatriatum surgical repair -a congenital heart disease-, the diagnosis of renal arteriovenous fistula remained insidious. The fistula was diagnosed during cardiac catheterization in an attempt to diagnose her rapidly decompensating heart failure, and repaired successfully by endovascular repair.

**Discussion:**

To our knowledge, there are only a few reports in literature describing iatrogenic renal artery-caval fistulas in association with laparoscopic cholecystectomy. Such high flow fistulas can result in a significant, potentially life threatening physiologic impairment. The case was managed by endovascular approach resulting in return to baseline cardiac function and resolution of symptoms.

**Conclusions:**

Renocaval arteriovenous fistulas are extremely rare to complicate laparoscopic cholecystectomy. It might go unnoticed, but may present with decompensated heart failure. It can be reversed by early recognition of symptoms, and diagnosis. High index of suspicion is a key, and endovascular modality is excellent treatment approach.

## Highlights

• Iatrogenic renal artery to inferior vena cava fistula formation is extremely uncommon after laparoscopic cholecystectomy.
• The chronic fistulas are usually insidious and diagnosed after an average period of 3- 25 years from the primary surgery.
• It can be challenging to diagnose these fistulas with the concomitant presence of cardiac diseases.
• High cardiac output fistulas such as renal-caval fistula can have devastating clinical consequences.
• Endovascular repair is a good approach, with less morbidity & mortality and provides excellent short and long term outcomes.

## 1 Introduction

Renal arteriovenous fistulas (RAVFs) may be congenital or acquired and are of the most common type of renal vascular abnormalities (70—80 %) [1], [11]. AVFs can exist almost anywhere in the body, depending on the aetiology. Acquired fistulas can be further subdivided into surgically created, as in for haemodialysis, or secondary to trauma, whether accidental or procedure related [11]. Most fistulas occur post-nephrectomy, angioplasty of the renal artery, blunt or penetrating trauma, nephrostomy, or percutaneous intervention for biopsies. Traumatic renal artery to inferior vena cava (IVC) fistulas are the most common type of acquired AVFs, and occasionally present with devastating clinical consequences, including hypertension, cardiomegaly, and high output heart failure. Early intervention, however, may deter complications of AVFs that can be avoided [12]. The goal of AVF treatment is to isolate and close the fistula while trying to keep essential renal blood flow. Repair may be completed by direct primary repair, open reconstruction (autogenous or prosthetic graft), or endovascular [13]. Endovascular treatment has become the first-line management of renal AVFs due to its lower morbidity and mortality, in addition to higher technical, clinical success rates, rapid recovery, less postoperative pain, and rapid return to normal daily activities compared with the open approach. The case was written in line with the SCARE guidelines [20].

## 2 Case presentation

A 43-year-old lady presented to cardiology services with progressively worsening shortness of breath in her-follow up visits. Her symptoms started following her laparoscopic cholecystectomy done a year ago complicated by bleeding, necessitating conversion to open surgery. She is a known case of congenital heart disease (cor-triatriatum repair 16 years ago; at the age of 27). She was on regular follow up with cardiology since then. She was doing fine after the congenital cardiac surgery operation. Her last follow-up prior to the laparoscopic surgery showed normal cardiac function, including LV ejection fraction of more than 55 %.

Her symptoms kept worsening despite medical treatment, turning into grade 3 (NYHA) heart failure, with exertional dyspnoea on minimal activity.

On physical examination, her heart rate was 68 bpm, with high blood pressure of 160/100 mmHg despite being on three antihypertensive medications of 100 mg Atenolol, 2.5 mg of Enalapril and 25 mg of Spironolactone all once daily. Cardiac exam revealed a pansystolic murmur. Jugular venous pressure was raised, with positive hepato-jugular reflux.

A 2-dimensional transthoracic echocardiogram revealed a mild to moderate eccentric mitral and tricuspid regurgitation. The right ventricular systolic pressure was elevated at 65-70 mmHg. Right atrial pressure was 20–25 mmHg with no evidence of a residual membrane in the left atrium. Her echocardiogram also revealed an EF to be more than 55 %. Her body-mass index was 21.7. Her laboratory tests, including thyroid function tests, arterial blood gas and renal function tests were normal, except for trace proteinuria (1+). She was eventually diagnosed with pulmonary hypertension.

Cardiac catheterization was done and showed no structural cardiac defect. During the same procedure, angiography showed an arterio-venous fistula between the inferior vena cava and renal artery. She was then referred to our vascular surgery clinic.

**Fig. 1:**
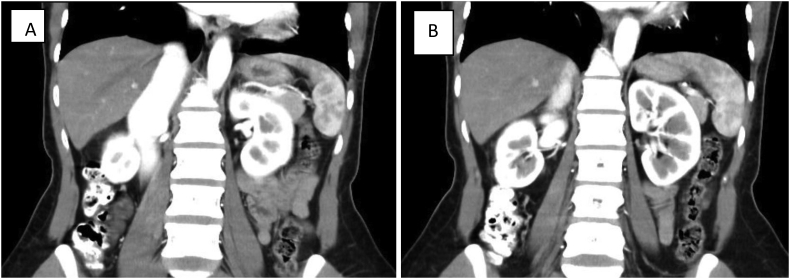
A and B Coronal reconstructions of CT angiography demonesrating flow through the renocaval fistula with contrast seen in the dilated inferior vena cava.

**Fig. 2:**
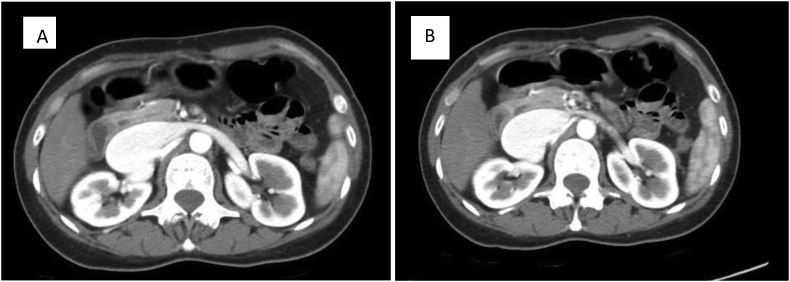
A and B: demonestrate CT angiography images in axial cuts, showing renovascular fistula with dilated contrast filled inferior vena cava.

We investigated her with a CT Angiography, it showed an arteriovenous fistula between the right renal artery and inferior vena cava (IVC). The fistula was found at 1.5 cm distal to the origin of the right renal artery. The IVC was significantly dilated with early filling (Fig. 1, Fig. 2). All findings were consistent with a high output heart failure secondary to a renocaval AVF. After explaining the procedure to the patient, an endovascular repair was planned.

## 3 Operative report

**Fig. 3:**
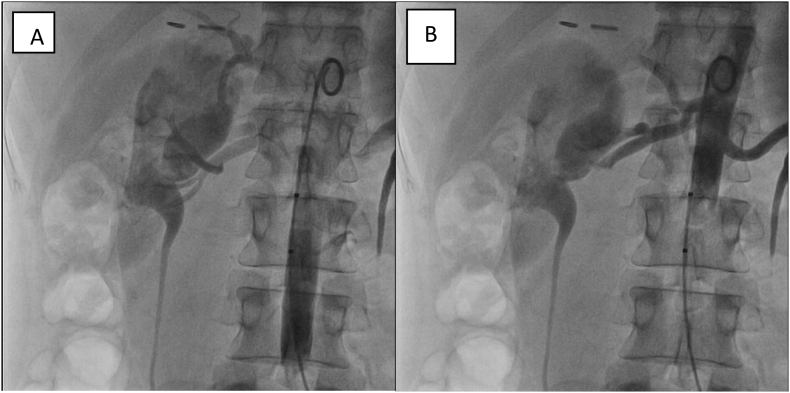
Intraoperative angiographic images depecting contrast escape from the renal artery to the dilated infferior vena cava.

Through a transfemoral approach, a 7-French sheath was inserted under local anaesthesia and right renal angiogram was performed which clearly showed a fistula between the right renal artery and inferior vena cava. The AVF was about 1.5 cm from the origin of the right renal artery with early IVC filling and poor renal parenchymal opacification (Fig. 3). A covered stent Gore® Viabahn ®, size 7 × 38 was deployed successfully. The completion angiogram confirmed optimum positioning of the endoprosthesis without any dissection and complete sealing of AVF (Fig. 3). She had a smooth postoperative course, and discharged home on the second post-operative day.

## 4 Follow up

**Fig. 4:**
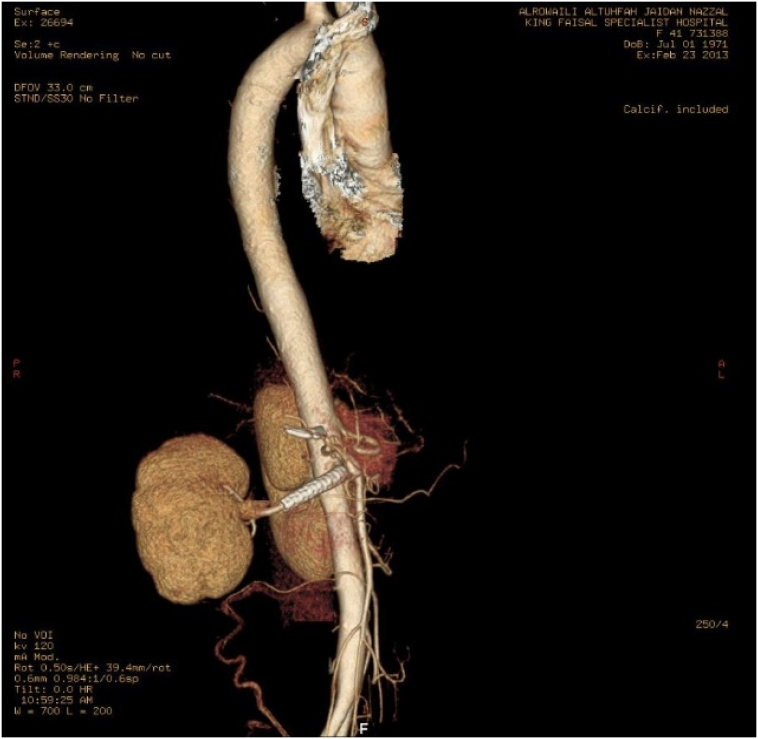
Post-operative 3D reconstruction of the right renal artery covered stent with absent flow to the vena cava following successful fistula covering.

The patient became completely symptom free, and not on any antihypertensive medication. Follow up echocardiography displayed normal left ventricular size, thickness, ejection fraction (50–55 %) and pressure. There was mild hypokinesia in the septum. The right atrium and ventricle were normal in function, and pressure. The left atrium, however, was severely dilated with no residual cor-triatriatum. All valves were normal in size and function except the mitral valve, which was thickened slightly, with no stenosis. There was no pericardial effusion. She was followed up annually for 8 years with a CTA or duplex ultrasound which showed a patent stent with no migration, and normal duplex hemodynamic of renal artery and vein [Fig. 4]. She remained asymptomatic.

## 5 Discussion

Traumatic AVFs are uncommon in post-operative setting [2]. Renal AVFs are exceedingly rarer. They are classified into acquired, idiopathic or congenital in the form of arterio-venous malformation. Frequency of iatrogenic AVFs increased, with the widespread use of percutaneous interventional procedures such as needle biopsies or percutaneous nephrostomy. Apart from interventional causes of traumatic renal AVFs, road traffic accidents, stab or projectile injuries can also cause traumatic AVFs. The incidence of such AVFs following percutaneous renal biopsy account for 1 to 18 %, out of which 39 % are mostly asymptomatic and resolve spontaneously (87 %) [1], [2]. Traumatic AVFs involving the IVC comprise 20 % of all abdominal AVF. If not repaired, they can lead to devastating outcomes, some of which include the development of chronic venous insufficiency/venous hypertension, high cardiac output failure with or without cardiomegaly and arterial insufficiency in some cases. The development of arteriovenous fistula leads to intractable heart failure via pressure gradient, size of the fistula and how long the fistula had been present. AVFs usually have high pulse pressure due to a decrease in diastolic blood pressure, which leads to a pulsatile arterial flow with decreased distal flow, thus compromising renal flow.

In our patient, she developed renal artery to-IVC fistula, which occurred as a complication of laparoscopic cholecystectomy. In the reported cholecystectomy, the operating surgeon encountered excessive bleeding, tried to identify where the bleeding site was, which eventually appeared to be from an artery originating from the gastroduodenal artery and he converted the procedure to open. With attempts to control the bleeding, however, and using multiple hemostatic tools including electrocautery, different biologic glues, the surgeon was able to achieve hemostasis. With background history of uncomplicated appendicectomy and cor-triatriatum repair for congenital heart disease more than 10 years prior to referral, she remained medically free until her presentation with hypertension and heart failure. Variations in hepatic and gallbladder blood supply are well documented in literature and can represent a surgical challenge to the unexperienced hands. Unlike the most common anatomy, where the cystic artery originates from the right hepatic artery, cystic artery was reported to originate with from the left hepatic, celiac trunk, pancreaticoduodenal, gastroduodenal, and superior mesenteric arteries. The presentation of AVF may vary depending on the location and aetiology. In severe, chronic, or high flow fistulas, patients can present with high output cardiac failure, resulting in shunting of oxygenated blood back to the right side of the heart. Due to the shortcut that the arterial blood takes through the venous system, this results in decreased peripheral resistance. In order to maintain blood pressure, the total circulating blood volume is increased, leading to heart failure. The Nicoladoni-Israel-Branham sign is a finding of reflex bradycardia with compression of the fistula due to increased afterload [3].

Table 1 summarizes the literature review on renal-inferior vena cava fistula as a complication of laparoscopic cholecystectomy. Renal-inferior vena cava fistula is mostly seen in middle-aged males. Di et al. reported signs of congestive heart failure, hypertension, and objective abdominal murmur and Lemmo et al. reported a history of chronic renal failure, systolic and diastolic hypertension, and congestive heart failure with cardiomegaly and chief complaint of acute dyspnoea [14], [15].

**Table 1:** Reported cases of revnal-caval arterio-venous fistulas.

Publish year	Author	Age	Sex	Intraoperative complications	Radiographic study type	Radiographic findings	Presentation	Number of year following the surgery
2001	Di	35	Man	Injury to the IVC with retro- peritoneal hematoma	Angiography	Revealed the arteriovenous fistula between the right renal artery and the IVC, which showed early arterial enhancement.	Signs of congestive heart failure, hypertension, and objective abdominal murmur classified as 4/6.	4 years
2002	Lemmo	33	Man	Bleeding from the vessels of the lesser gastric curvature and from the peripancreatic vessels, hypotension and tachycardia.	Angiography	Presence of a pseudoaneurysm of the right A. The IC was opacified through a fistula from the pseudoaneurysm of the right RA and appeared twofold in size.	Chronic renal failure, systolic and diastolic hypertension, and congestive heart failure with cardiomegaly	5 years

Timeline:

In the described case, the patient presented with symptoms of right-sided heart failure, along with pulmonary hypertension. The diagnosis remained insidious for three years because the presentation of high output fistula and cor-triatriatum (congenital cardiac defect with mechanical membrane obstruction of atrium) mimicking each other. Cor-triatrium is one of the rarest congenital cardiac defects, first described by Church, and clinically diagnosed in only 0.1 % of patients. It is characterized by presence of three atria in the heart, due to a fibro-muscular membrane giving rise to an accessory chamber in the left atrium, most cases are found after autopsy (0.4 %). It is usually associated with ASD and total anomalous pulmonary drainage to the right atrium, bicuspid aortic valve, tetralogy of Fallot and double outlet right ventricle [4], [5], [6].

While digital substraction angiography being the gold standard for diagnosis and valuable therapeutic tool, computed tomography angiography is an excellent, non-invasive modality to diagnose vascular anomalies, identification of sources of bleeding and anatomical variations, size and extent of iatrogenic A-V fistulas and surgical planning with sensitivities approaching 90 %.

There is little data available about the effect treating arteriovenous fistulas on hypertension. A review of 17 hypertensive patients with congenital A-V malformations and 14 hypertensives with traumatic lesions showed that the improvement in BP after fistula embolization occurred more frequently in traumatic fistulas than in congenital counterparts. In another review, hypertension associated with arteriovenous malformations was cured in 62 % of cases after surgical treatment [7], [8], [9].

AVFs are preferably diagnosed initially using a Doppler ultrasound showing hypoechoic cystic or tubular structures, which may be confused with renal cysts or hydronephrosis. Computed tomography of the abdomen would reveal tortuous, thinly dilated draining renal veins. MRI would reveal serpiginous flow-related signals. Angiography is the gold standard and has shown superior accuracy over CTA. It is the most invasive evaluation of an AVF but provides the exact point of arteriovenous communication surrounding vascular anatomy, and flow dynamics [14], [15], [16].

They can be managed by either endovascular or surgical approach. The decision of which approach to be adopted largely depends on the level of surgical expertise, available resources and hemodynamic status of the patient. In one review, a patient sustained penetrating injury to the renal artery and IVC presented with hemodynamic instability. In that case, the patient was managed surgically with excellent outcome [10], [18], [19].

## 6 Conclusion

Iatrogenic renal artery to inferior vena fistula is extremely uncommon after laparoscopic cholecystectomy. The chronic fistulas are usually insidious and diagnosed after an average period of 3–25 years. Sometimes, it is challenging to diagnose these fistulas with the concomitant presence of cardiac diseases. High cardiac output fistulas such as renal-caval fistula can have devastating clinical consequences, including development of hypertension and congestive heart failure. Angiography is the gold standard for diagnosis. Endovascular technique is preferred to surgery due to its less invasive nature, lower morbidity and mortality and good short and long-term outcomes [11].

## Consent

All information used in this case including pictures were published after obtaining an informed consent.

## Ethical approval

The case was reported in accordance with the ethical committee report meeting at King Faisal specialist hospital and Research center with RAC# 2225274.

## Funding

No funding was received for this publication.

## Author contribution

Ahmed Almumtin: Concept, literature review, and writing.

Mohammed Dahman: Literature review and revisions.

Bassam Khalil: Literature review and data collection.

Elaf Almosaihel: Literature review and data collection.

Maitham Alabduljabbar: Literature review and revision.

Samer Koussayer: Overall supervision, consultant surgeon on the case.

## Guarantor

Ahmed Almumtin.

## Registration of research studies

None.

## Declaration of competing interest

No conflict of interest.
